# Ornithine uptake and the modulation of drug sensitivity in *Trypanosoma brucei*

**DOI:** 10.1096/fj.201700311R

**Published:** 2017-07-05

**Authors:** Juan P. Macedo, Rachel B. Currier, Corina Wirdnam, David Horn, Sam Alsford, Doris Rentsch

**Affiliations:** * Institute of Plant Sciences, University of Bern, Bern, Switzerland;; † London School of Hygiene and Tropical Medicine, London, United Kingdom;; ‡ Wellcome Trust Centre for Anti-Infectives Research, School of Life Sciences, University of Dundee, Dundee, United Kingdom

**Keywords:** African trypanosomiasis, chemotherapy, eflornithine, suramin, histidine

## Abstract

*Trypanosoma brucei*, protozoan parasites that cause human African trypanosomiasis (HAT), depend on ornithine uptake and metabolism by ornithine decarboxylase (ODC) for survival. Indeed, ODC is the target of the WHO “essential medicine” eflornithine, which is antagonistic to another anti-HAT drug, suramin. Thus, ornithine uptake has important consequences in *T. brucei*, but the transporters have not been identified. We describe these amino acid transporters (AATs). In a heterologous expression system, TbAAT10-1 is selective for ornithine, whereas TbAAT2-4 transports both ornithine and histidine. These AATs are also necessary to maintain intracellular ornithine and polyamine levels in *T. brucei*, thereby decreasing sensitivity to eflornithine and increasing sensitivity to suramin. Consistent with competition for histidine, high extracellular concentrations of this amino acid phenocopied a TbAAT2-4 genetic defect. Our findings established TbAAT10-1 and TbAAT2-4 as the parasite ornithine transporters, one of which can be modulated by histidine, but both of which affect sensitivity to important anti-HAT drugs.—Macedo, J. P., Currier, R. B., Wirdnam, C., Horn, D., Alsford, S., Rentsch, D. Ornithine uptake and the modulation of drug sensitivity in *Trypanosoma brucei*.

*Trypanosoma brucei* is a vector-borne protozoan parasite and the causative agent of African trypanosomiasis, which includes human and animal diseases endemic in 37 countries in sub-Saharan Africa. The subspecies *T. b. gambiense* and *T. b. rhodesiense* cause human African trypanosomiasis (HAT), also known as sleeping sickness, which is typically fatal if untreated. *T. b. brucei* and the related species *T. congolense* and *T. vivax* cause nagana, a wasting disease of cattle that is a major obstacle to the economic development of affected rural areas (1). The number of reported cases of HAT is currently ∼4000 per year, but it is estimated that the true figure is closer to 20,000 (1, 2). The pathology is divided into two stages. The first stage is characterized by the proliferation of the parasite in the blood and lymph, whereas in the second stage the parasites invade the cerebrospinal fluid and the brain, causing confusion, an altered sleep–wake pattern, and ultimately, lethal coma (3). Chemotherapy against the first stage of HAT is based on pentamidine or suramin, whereas the second stage can be treated with the organoarsenical compound melarsoprol, which is associated with severe adverse effects, or the nifurtimox/eflornithine combination therapy (3), which is currently the treatment of choice for *T. b. gambiense*, but is not recommended in infections caused by *T. b. rhodesiense* because of the lower innate susceptibility of this subspecies to eflornithine (4).

Eflornithine, taken up by the neutral amino acid transporter TbAAT6 (5–8), is a well-known suicide inhibitor of ornithine decarboxylase (ODC) (9), a key enzyme in the polyamine biosynthetic pathway (**Fig. 1**). Polyamines are small cationic molecules essential in eukaryotic cells and most bacteria (10). In the cell, they interact with RNA and proteins, modulating gene expression and cell growth (11). A universal function of polyamines is, for example, the deoxyhypusine modification of eukaryotic initiation factor 5A (eIF5A) (12, 13). In *T. brucei*, polyamines are precursors for the synthesis of trypanothione, a trypanosomatid-specific thiol that has an essential role in redox regulation and defense against oxidative damage (14) and is associated with drug extrusion in the related trypanosomatid *Leishmania* (15). Polyamines are predominantly derived from the amino acids ornithine and methionine. Ornithine is decarboxylated into putrescine by ODC. Putrescine is then converted into spermidine by addition of aminopropyl groups donated by decarboxylated *S*-adenosylmethionine. Subsequently, spermidine is combined with two molecules of glutathione to form trypanothione (16). Eflornithine treatment of bloodstream-form (BSF) *T. brucei* leads to reduced intracellular putrescine, spermidine, and trypanothione levels (17, 18). Suramin action, on the other hand, is potentiated by the polyamine biosynthetic pathway (19); depletion or inhibition of ODC or depletion of other spermidine biosynthetic enzymes rendered BSF parasites less sensitive to this drug (19).

**Figure 1.**
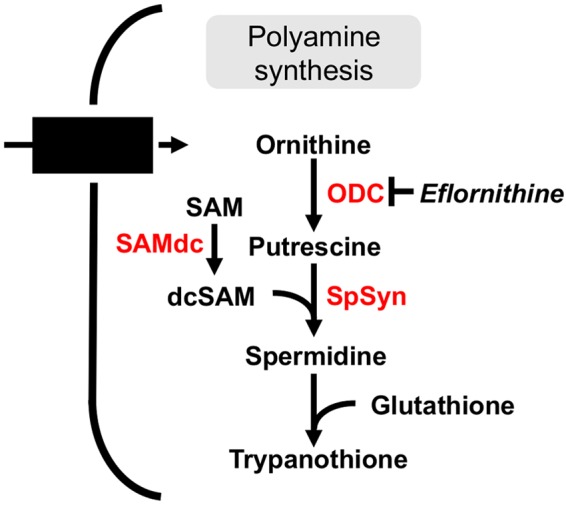
The spermidine–trypanothione biosynthetic pathway. Down-regulation of spermidine synthesis leads to reduced suramin efficacy (19). ODC, ornithine decarboxylase; SAM, *S*-adenosylmethionine; dcSAM, decarboxylated *S*-adenosylmethionine; SAMdc, *S*-adenosylmethionine decarboxylase; SpSyn, spermidine synthase.

The polyamine biosynthetic pathway is ubiquitous and shows a high degree of conservation across the Eukarya; however there are some notable variations (20). For example, *Leishmania* can use arginase for the *de novo* production of ornithine from arginine (21). This trypanosomatid also has the capacity for high-affinity putrescine and spermidine uptake (22). *Trypanosoma cruzi* lacks a functional ODC (23, 24) and relies on high-affinity putrescine/cadaverine uptake or the spermidine transport system for polyamine acquisition (25, 26). In contrast, ODC activity in *T. brucei* is essential, as indicated by the parasite’s susceptibility to eflornithine and ODC knockdown experiments (3, 27, 28); however, supplementation with putrescine renders ODC dispensable, suggesting a putrescine uptake system in *T. brucei* (29). Unlike *T. cruzi*, *T. brucei* is unable to take up sufficient spermidine from its environment when polyamine biosynthesis is disrupted, indicating that the African trypanosome lacks an efficient spermidine transporter (30). Finally, *T. brucei* lacks a canonical arginase; instead, it possesses an arginase-like protein that is unable to convert arginine into ornithine (31, 32). A recent metabolomic analysis revealed that *T. brucei* is capable of converting arginine to ornithine by an unknown mechanism, but its principal source of ornithine comes *via* uptake from the environment (33), supporting the hypothesis that *T. brucei* is auxotrophic for ornithine, and therefore, is reliant on ornithine import for polyamine biosynthesis.

In this study, we report the functional characterization of two members of the amino acid transporter (AAT) family, one of which has been implicated in suramin action. These AAT family members are novel high-affinity ornithine transporters, playing an essential role in the mammalian life-cycle stage of *T. brucei* and therefore represent the key uptake systems for polyamine precursors. Notably, reduction in ornithine transport renders BSF *T. brucei* hypersensitive to eflornithine.

## MATERIALS AND METHODS

### *T. brucei* lines

BSF *T. brucei* MITat1.2/2T1 (34) and New York single-marker (NY-SM) (35) coexpressing T7 RNA polymerase and a tetracycline (tet) repressor were cultured at 37°C in HMI-11 containing 10% (v/v) heat-inactivated fetal bovine serum (FBS). Creek’s minimal medium (CMM) was prepared (36) with slight modifications: containing 10% (v/v) heat-inactivated FBS and 0.1 mM tyrosine, phenylalanine, tryptophan, leucine, methionine, arginine, and hypoxanthine. Ornithine comes exclusively from FBS and was described to be 25–45 µM in HMI-11 and 15–20 µM in CMM (37).

### RNA interference constructs

RNA interference (RNAi) target fragments were designed using the RNAit primer design tool (38), and PCR amplified from *T. brucei* genomic DNA with the following primers: TbAAT10-1xb, 5′-GATCTCTAGAGGATCCTCGTGTCTAAATGGGCTTCC-3′, and TbAAT10-1ba, 5′-GATCGGGCCCGGTACCCTTTGGGATGAAGAGACCCA-3′ (spanning nt 691–1206 of Tb427.08.8290 for pRPa^iSL^-AAT10-1); TbAAT10-1RNAiF 5′-GGCCAAGCTTGGATCCGGATGATGGCATTAAAAACTATG-3′ and 5′-GGCCTCTAGACTCGAGCA CGTAGGCAATACAACTCG-3′ (spanning nt 75–477 of Tb427.08.8290 for pALC14-AAT10-1) for selective down-regulation of TbAAT10-1; TbAAT2-4RNAiF 5′-GGCCAAGCTTGGATCCATTTGCTGCATTCATCCCTC-3′ and TbAAT2-4RNAiR 5′-GGCCTCTAGACTCGAGGCATAAAACATGCCCAAACC-3′ (spanning nt 168–592 of Tb427.04.4020 for pMS14-AAT2-4); and TbAAT10-2RNAiF 5′-GCCCAAGCTTGGATCCAGGTGAGTTTATGCATCGCC-3′ and TbAAT10-2RNAiR 5′-TGGCTCTAGACTCGAGCAAGACGCGGTTACCTGATT-3′ (spanning nt 155–565 of Tb427.08.8300 for pMS14-AAT10-2). Appropriate restriction sites (underlined) were incorporated into the primers to enable 2-step cloning into the stem-loop RNAi plasmids pRPa^iSL^ (TbAAT10-1) (39), pALC14 (TbAAT10-1), and pMS14 (TbAAT10-2 and TbAAT2-4) (35, 40). pRPa^iSL^-TbAAT10-1 was linearized with *Asc*I to enable targeted integration into the landing pad locus in 2T1 strain *T. brucei* (34, 39). pALC14 and pMS14 stem-loop RNAi plasmids were linearized with *Not*I before transfection.

### Stable transfection

2T1 and NY-SM *T. brucei* were transfected as described elsewhere (7, 34). In brief, trypanosomes were harvested at mid log phase and washed once in PBS (pH 7). 2T1 and NY-SM *T. brucei* were respectively resuspended in 100 μl cytomix or Tb-BSF buffer (7) containing ∼10 µg of DNA. Electroporation was performed in 0.2-mm-gap cuvettes with a Nucleofector (2T1 *T. brucei*) or 4D Nucleofector System (NY-SM *T. brucei*; Lonza, Basel, Switzerland), using program X-001 and FI-115, respectively. Transfected cells were immediately inoculated in culture medium and distributed across multiwell plates. After at least 5 h, the appropriate selective antibiotics were added [*i.e.*, 2.5 μg/ml hygromycin (pRPa^iSL^), 2.5 μg/ml phleomycin (pMS14), or 0.1 μg/ml puromycin (pALC14)]. Clonal transformants were identified after at least 5 d in culture, and 2T1/pRPa^iSL^ landing pad integration was confirmed by assessing puromycin sensitivity (34).

For the TbAAT2-4/TbAAT10-1 double-RNAi line, TbAAT2-4 RNAi BSF was transfected with *Not*I-linearized TbAAT10-1-pALC14 construct and selected in 0.1 µg/ml puromycin. All subsequent growth and drug sensitivity assays were performed in the absence of selective antibiotics.

### Drug sensitivity assay

Susceptibility of BSF *T. brucei* to eflornithine and suramin was assessed in 96-well plates (41). In brief, serial dilutions (1:2) of eflornithine or suramin were prepared in HMI-11 medium containing 10% (v/v) FBS. An equal volume of parasite suspension was added to each well to a final density of 1 × 10^4^ cells/ml; RNAi was preinduced for at least 24 h in tet, and then induction was maintained throughout the assay. After 70 h incubation at 37°C, 125 µg/ml resazurin in PBS (pH 7) was added to a final concentration of 12.5 µg/ml, and incubation was continued for another 2 h at 37°C. Fluorescence was measured with a M200 plate reader (Tecan, Männedorf, Switzerland) at 544 nm excitation and 590 nm emission, with gain optimization. EC_50_ values were derived from dose–response curves (variable slope) in Prism 6.0 software (GraphPad, La Jolla, CA, USA).

### Quantitative RT-PCR

Total RNA was isolated with the SV RNA isolation system (Promega, Madison, WI, USA) according to the manufacturer’s instructions. RNA samples were treated with DNase I (Roche, Basel, Switzerland) for 25 min at 37°C, followed by phenol/chloroform extraction and ethanol precipitation. Absence of genomic DNA contamination was confirmed by PCR. DNase I-treated RNA (0.5 µg) was used for cDNA synthesis with PrimeScript reverse transcriptase (Takara, Shiga, Japan). Real-time quantitative PCR (qPCR) was performed with a LightCycler 480 System (Roche). The reaction mixtures consisted of 1× Sybr green premix, Ex Taq (RR420L; Takara), and 0.2 µM primers (TbAAT10-1qPCR_F, 5′-TTCAGTGGGAGATATTTTCTCTTCT-3′, and TbAAT10-1qPCR_R, 5′-CCATAGAAGCGCGGTCAAAAG-3′; TbAAT2-4qPCR_F, 5′-CAGGTAATCGTCTTCTCACG-3′, and TbAAT2-4qPCR_R, 5′-GGCAAAAGTGGAGACGTAC-3′; and TbAAT10-2qPCR_F, 5′-ATCTTTGGGGGATATTATGTCTTCT-3′, and TbAAT10-2qPCR_R, 5′-ATTACCGTCAAGACGCGGTT-3′). Real-time PCR analyses were performed with 3 different cDNA dilutions. Telomerase reverse transcriptase (Tb927.11.10190) was used as the reference gene (42).

### *Saccharomyces cerevisiae* transformation

For expression of TbAAT2-4, TbAAT10-1, and TbAAT10-2 in *Saccharomyces cerevisiae*, the open reading frames were PCR amplified from *T. brucei* strain 427 genomic DNA and cloned into the yeast expression vector, pDR197 (43), using the primers: TbAAT2-4ORF_F, 5′-CGGAATTCATGTGTATTGCCAGAGAAAATACAAGC-3′, and TbAAT2-4ORF_R, 5′-CGCGGATCCTTAACCAGTTATAGTGCCCCATATAC-3′; TbAAT10-1ORF_F, 5′-CGGAATTCATGAGTAATGTCCGTGGAAATATAACCC-3′, and TbAAT10-1ORF_R, 5′-CGCGGATCCTTAGCCAACAATTGTGCCCCAAATG-3′; and TbAAT10-2ORF_F, 5′-CGGAATTCATGGACAGATCTGGCAGTCAATCCGC-3′, and TbAAT10-2ORF_R, 5′-CGCGGATCCTCAACCCACAGCAGCGCCCC-3′. Appropriate restriction sites (underlined) were included in the primer sequences. Independently amplified open reading frames were confirmed by sequencing and compared with predicted open reading frames in TritrypDB (*http://tritrypdb.org/tritrypdb/*). Transformation of *S. cerevisiae* was performed according to Dohmen *et al.* (44). The *S. cerevisiae* mutant JT16 (*Mata*, *hip1*-*614*, *his4*-*401*, *can1*, *ino1*, *ura3*-52 (45) is auxotrophic for histidine and has reduced histidine transport rates provided the general amino acid permease is down-regulated in the presence of ammonium; 22∆8AA (*MATα*, *gap1*-*1*, *put4*-*1*, *uga4*-*1*, *lyp1/alp1::hisG*, *can1::hisG*, *hip1::hisG*, *dip5::hisG*, *ura3*-*1* (46) carries mutations in the major uptake systems for proline, GABA, citrulline, arginine, lysine, histidine, and glutamate/aspartate; 22∆6AAL (*Matα*, *ura3*-*1*, *gap1*-*1*, *put4*-*1*, *uga4*-*1*, *can1::hisG*, *lyp1/alp1::hisG*, *lys2::hisG* (46) is lacking the major uptake systems for proline, GABA, citrulline, arginine, and lysine, and is auxotrophic for lysine. Additional strains and media for selective and nonselective growth were as described in Mathieu *et al*. (8). For transport experiments using strain 22∆8AA, transformed cells were grown in synthetic dextrose minimal medium [sd: 1.7 g/L yeast nitrogen base without amino acids and without ammonium sulfate (Difco; Becton Dickinson, Sparks, MD, USA), 5 g/L ammonium sulfate, and 20 g/L glucose].

### Transport assays

Transport assays using the *S. cerevisiae* strain 22Δ8AA were performed as described (47), with slight modification. Cells were grown to a density of OD_600_ 0.6, washed twice with water, and resuspended in buffer A [1:10 initial volume; 0.6 M sorbitol and 50 mM potassium phosphate, (pH 5.5) adjusted with KOH]. Before the transport assay, cells were preincubated at 30°C for 5 min in the presence of 100 mM glucose. To start the transport assay, cells (130 µl) were added to an equal volume of buffer with different concentrations of l-ornithine or l-histidine and 7.2 kBq l-[^3^H]ornithine or l-[^3^H]histidine (2.2 and 1.7 TBq/mmol; Hartmann Analytic, Braunschweig, Germany) per assay. In some experiments, competitors were added, as specified in the Results section.

Samples (48 µl) were transferred after 30 s and 1, 2, 3, and 5 min to 4 ml ice-cold buffer A, filtrated on glass fiber filters and washed twice with 4 ml ice-cold buffer A. The uptake of tritium-labeled substrates was determined by liquid scintillation spectrometry, and transport rates were calculated. Uptake rates by *S. cerevisiae* transformed with empty vector were subtracted as background. Kinetic parameters were calculated with the Michaelis-Menten equation, *V* = *V*_max_ × [*S*] × (*K*_m_ + [*S*])^−1^, in Prism 6.0 (GraphPad).

### Amino acid and polyamine analysis

Parasites (∼3 × 10^7^ cells) collected from cultures grown to mid log phase were harvested, and metabolites were extracted in 200 µl of chloroform:methanol:water (1:3:1) (48). Analysis of amino acids was based on a method described elsewhere (49). The free amino acids were labeled with phenylisothiocyanate. The resulting phenylthiocarbamoyl amino acids were separated by RP-HPLC on a Nova Pak C18 column (3.9 × 150 mm 4 µm; Waters, Milford, MA, USA) in a Summit liquid chromatograph (Dionex, Sunnyvale, CA, USA) and monitored by UV detection at 247 nm. A gradient of 2–60% acetonitrile (60% v/v) from 0–13 min and 60–100% from 13 to 26 min was applied. For the elution of polyamines, the gradient was extended to 26 min.

## RESULTS

### The putative amino acid transporter TbAAT10-1 (Tb427.08.8290) shows opposing effects on suramin and eflornithine efficacy

Suramin selection of the BSF *T. brucei* RNAi library and subsequent RIT-seq mapping against the genome of *T. brucei* strain TREU927 (19, 50) identified not only proteins involved in drug uptake, endolysosomal function, and polyamine biosynthesis, but also several putative AATs belonging to the amino acid/auxin permease family (51). The only amino acid transporter to fulfill our established stringency criteria [>99 reads and 2 or more independent RNAi target fragments (50)] was Tb427.08.8290 (TbAAT10-1), a member of the AAT10 subgroup (19, 52). To validate this finding, we generated 2T1 *T. brucei* stem-loop RNAi strains targeting TbAAT10-1.

**Figure 2.**
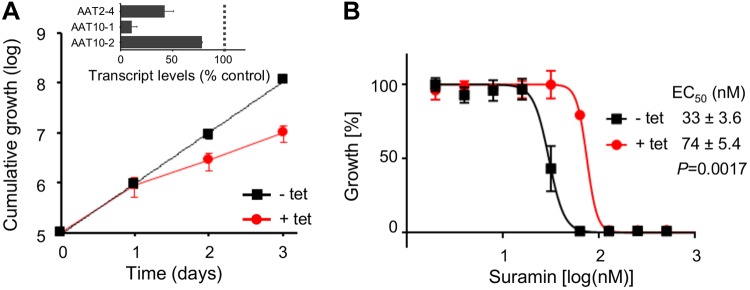
TbAAT10-1 RNAi impacts growth and suramin sensitivity of BSF *T. brucei*. *A*) Cumulative growth of BSF *T. brucei* after TbAAT10-1 RNAi in the presence of 1 µg/ml tet; mean of 2 independent clones. Error bars denote sd. Inset: real-time qPCR analysis showing TbAAT mRNA depletion after 24 h induction in 1 µg/ml tet. Outputs were normalized to telomerase reverse transcriptase expression and are shown as a percentage of uninduced cells. Data from at least 2 independent clones per target are shown. Error bars denote sem. *B*) Representative quadruplicate EC_50_ assay showing the effect of TbAAT10-1 depletion on suramin efficacy against *T. brucei*. RNAi was induced in 2.5 ng/ml tet 24 h before drugs were added and throughout the experiment. Error bars denote sd. Inset: summary statistics from 3 independent clones. *P* values obtained by paired Student’s *t* test.

TbAAT10-1 depletion by RNAi in 1 µg/ml tet led to a growth defect under culture conditions (**Fig. 2*A***), whereas partial induction in 2.5 ng/ml tet [to minimize the impact on parasite growth during the 4 d assay (19, 53)] resulted in an ∼2-fold increase in suramin EC_50_ (Fig. 2*B*), supporting a role for TbAAT10-1 in determining suramin efficacy. However, real-time qPCR analysis revealed that the expression of the related transporter Tb427.04.4020 (TbAAT2-4) was down-regulated by ∼50% (subsequently referred to as TbAAT10-1/2-4 RNAi; Fig. 2*A* inset). TbAAT2-4, a member of the *TbAAT2* locus, is a close homolog of TbAAT10-1, as is Tb427.08.8300 (TbAAT10-2; Supplemental Tables 1 and 2). In fact, although the *TbAAT10* locus contains 2 members, TbAAT10-1 and TbAAT10-2, the closest homolog of TbAAT10-1 is TbAAT2-4 (Supplemental Table 2). The sequences of the 3 AATs were confirmed by sequencing and alignment with the genome sequences of *T. brucei* TREU 927 and Lister 427, the latter including several unresolved nucleotides in Tb427.4.4020 and Tb427.08.8290 (data not shown; the resolved sequences have been uploaded to www.TriTrypDB.org and associated with the corresponding gene pages). All subsequent sequence analysis in the present article is based on these confirmed sequences from Lister 427, our experimental strain.

**Figure 3.**
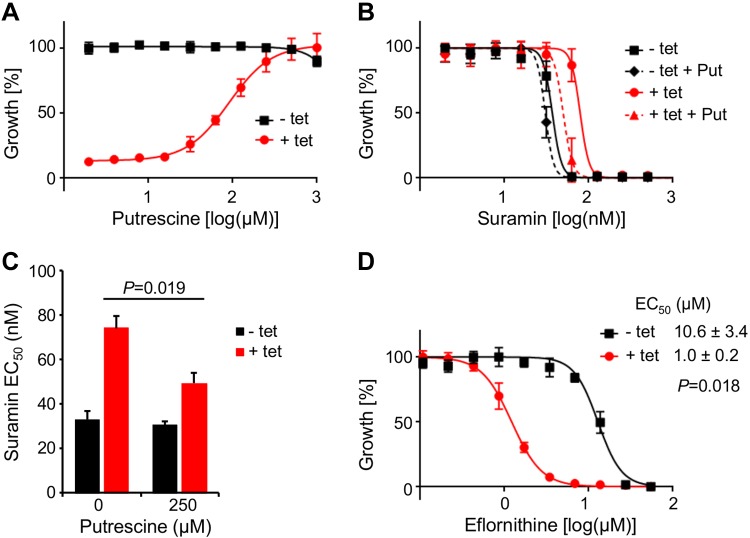
TbAAT10-1/2-4 RNAi phenotypes are complemented by supplementation with the spermidine pathway intermediate putrescine (Put), whereas down-regulation increases eflornithine sensitivity. *A*) The effect of putrescine supplementation on growth after RNAi against TbAAT10-1/2-4; RNAi induced in 1 µg/ml tet for 72 h in the presence of putrescine (2 µM–1 mM). The assay was performed in quadruplicate and visualized after addition of 12.5 µg/ml resazurin. Growth is represented as a percentage of cell growth in the absence of tet and putrescine. Error bars denote sd. Three independent TbAAT10-1/2-4 RNAi clones gave comparable results; a representative example is shown. *B*) Representative quadruplicate EC_50_ assay showing the effect of supplementation with 250 µM putrescine on suramin efficacy after TbAAT10-1/2-4 knockdown. RNAi was induced in 2.5 ng/ml tet 24 h before addition of suramin and throughout the experiment. *C*) Pooled EC_50_ data for 3 independent clones showing the impact of putrescine supplementation on suramin efficacy after TbAAT10/2-4 RNAi. Error bars denote sd. *P* value (+tet *vs.* +tet+put) derived by paired Student’s *t* test. *D*) Representative quadruplicate EC_50_ assay showing the effect of TbAAT10-1/2-4 RNAi on eflornithine efficacy against *T. brucei*; RNAi induced in 2.5 ng/ml tet 24 h before drug addition and throughout the experiment. Error bars denote sd. Inset: summary statistics from 3 independent clones. *P* values derived by paired Student’s *t* test.

The small, though significant, shift in suramin EC_50_ seemed unlikely to be related to a direct interaction with suramin, but instead pointed to an indirect effect, possibly resulting from an impact on lysosomal function, spermidine synthesis, or both. As has been demonstrated, down-regulation of key enzymes in the spermidine biosynthetic pathway, including ornithine decarboxylase, spermidine synthase, and *S*-adenosylmethionine decarboxylase (see Fig. 1) also reduces suramin efficacy (19). We therefore hypothesized that TbAAT10-1 transports an amino acid that feeds into the spermidine biosynthetic pathway. Supplementation of the culture medium with putrescine progressively reduced the growth phenotype associated with TbAAT10-1/2-4 RNAi (**Fig. 3*A***), and supplementation with 250 µM putrescine partially complemented the suramin resistance phenotype (Fig. 3*B*, *C*). The high putrescine concentrations necessary for complementation indicate that putrescine uptake by *T. brucei* is not efficient, further emphasizing the importance of polyamine biosynthesis and the fundamental role of ODC in these parasites (27, 28). The connection to the spermidine biosynthetic pathway, as well as the absence of a canonical arginase in *T. brucei* (31, 33), supports a role in ornithine import. Consistent with this hypothesis, induction of TbAAT10-1/2-4 RNAi led to an ∼10-fold increase in parasite sensitivity to eflornithine compared to that of noninduced cells (Fig. 3*D*).

**Figure 4.**
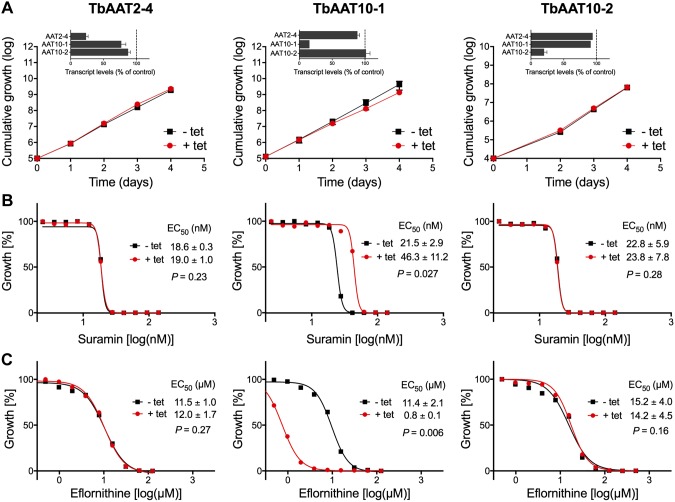
Decreased suramin and increased eflornithine efficacy are also observed upon specific knockdown of TbAAT10-1 in BSF *T. brucei*. *A*) Cumulative growth of BSF *T. brucei* after depletion of TbAAT2-4, TbAAT10-1, or TbAAT10-2. RNAi induced in 1 µg/ml tet. Data points are means ± sd from 3 technical replicates. Insets: transcript levels determined by qPCR, shown as a percentage of uninduced cells and normalized to telomerase reverse transcriptase expression. Means ± sem from at least 2 technical replicates. *B*, *C*) Representative triplicate EC_50_ assays showing the impact of TbAAT2-4, TbAAT10-1, and TbAAT10-2 RNAi on BSF *T. brucei* sensitivity to suramin(*B*) and eflornithine (*C*). RNAi induced in 1 µg/ml tet 24 h before drug addition and throughout the experiment. Data points are means ± sem of a representative experiment performed in triplicate. Insets, EC_50_ ± sd from 3 independent experiments. *P* values derived by paired Student’s *t* test.

Suramin selection of the BSF *T. brucei* RNAi library implicated TbAAT10-1 knockdown in eliciting the observed growth defect and changes in suramin and eflornithine efficacy, but TbAAT2-4 was also knocked down in these experiments (Fig. 2*A*). To assess the contributions of each transporter individually, we used distinct regions to generate BSF *T. brucei* RNAi lines showing specific down-regulation of TbAAT10-1, TbAAT2-4, or TbAAT10-2 (Supplemental Fig. 1). Specific down-regulation of TbAAT10-1 in 1 µg/ml tet only marginally impaired growth (**Fig. 4*A***). However, this TbAAT10-1 specific knockdown reduced sensitivity to suramin ∼2-fold (Fig. 4*B*) and increased sensitivity to eflornithine ∼10-fold (Fig. 4*C*). In contrast, specific down-regulation of TbAAT2-4 or TbAAT10-2 had no effect on growth (Fig. 4*A*), suramin sensitivity (Fig. 4*B*), or eflornithine efficacy (Fig. 4*C*). These data indicate that down-regulation of TbAAT10-1 alone in BSF *T. brucei* is sufficient to confer reduced sensitivity to suramin and to enhance eflornithine efficacy, whereas the growth defect is more pronounced when both TbAAT10-1 and TbAAT2-4 are down-regulated (compare Figs. 2*A* and 4*A*). To determine whether the growth phenotype observed in Fig. 2*A* was related to down-regulation of TbAAT10-1 combined with substantial down-regulation of TbAAT2-4, we transfected the TbAAT2-4-specific RNAi cell line with the TbAAT10-1-specific stem-loop RNAi construct. This double RNAi cell line exhibited similar TbAAT10-1/2-4 down-regulation efficiency and a similar growth defect (Supplemental Fig. 2) when compared to the original TbAAT10-1/2-4 RNAi cell line (Fig. 2*A*). Furthermore, the growth arrest observed following specific TbAAT2-4/TbAAT10-1 double RNAi could be rescued by the addition of high (nonphysiologic) concentrations of ornithine, supporting the hypothesis that ornithine import is compromised in these cells, but that there are additional low-affinity ornithine uptake systems (Supplemental Fig. 2).

### TbAAT10-1 and TbAAT2-4 are high-affinity transporters for ornithine and ornithine/histidine, respectively

**Figure 5.**
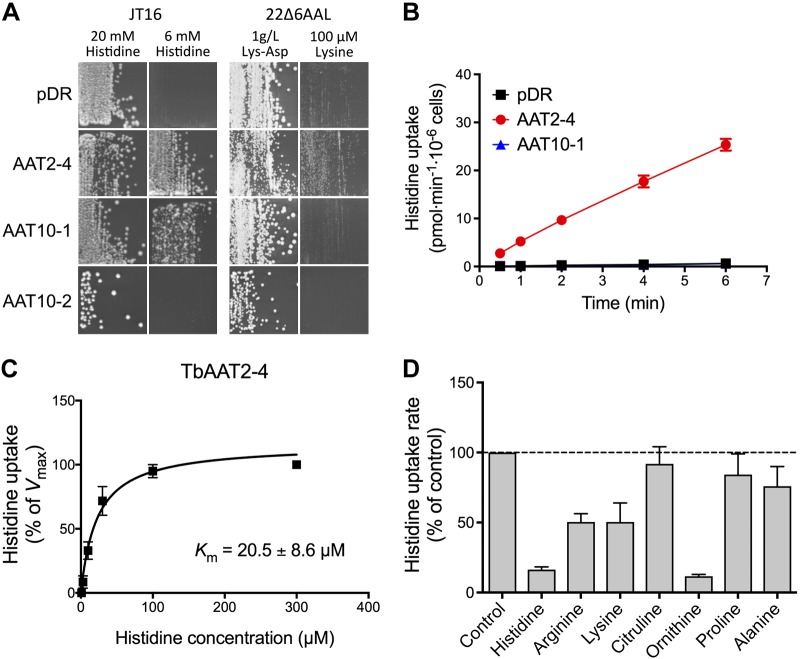
TbAAT2-4 and TbAAT10-1 mediate growth of *S. cerevisiae* mutants on histidine. *A*) *S. cerevisiae* mutants transformed with the vector pDR197 (pDR) or pDR197 harboring TbAAT2-4, TbAAT10-1, or TbAAT10-2 were grown for 3 d on nonselective (left) and selective (right) media. JT16 and 22∆6AAL strains allow selection for histidine and lysine uptake, respectively. The dipeptide Lys-Asp was used as a source of lysine in the nonselective medium for strain 22∆6AAL. *B*) Time-dependent 5 µM histidine uptake by *S. cerevisiae* (strain 22∆8AA) transformed with vector (pDR) or expressing TbAAT2-4 or TbAAT10-1. Data points are means ± sem from 2 independent experiments. *C*) Transport kinetics of *S. cerevisiae* expressing TbAAT2-4. Uptake rates were normalized to maximum transport rates (*V*_max_; *i.e.*, from 15.7 to 30 pmol/min/10^6^ cells). Data points and apparent affinities (*K*_m_) are means ± sd from 4 independent experiments. *D*) TbAAT2-4-mediated histidine uptake (200 µM histidine) in the absence (control) or presence of the indicated compounds (2 mM). Histidine transport rates were determined at 5 time points: 30 s and 1, 2, 3, and 5 min. Data sets were normalized to control uptake rates (15–19 pmol/min/10^6^ cells) and are means ± sd from 3 independent experiments.

The role of these amino acid transporters in the uptake of ornithine and other amino acids was tested by heterologous expression in *S. cerevisiae* mutants. Although the *S. cerevisiae* mutants tested did not allow us to assess the transporters’ ability to support growth on ornithine, these experiments revealed that TbAAT10-1 is able to support growth of *S. cerevisiae* strain JT16 on histidine, whereas TbAAT2-4 supported growth on histidine and lysine (strains JT16 and 22∆6AAL, respectively, **Fig. 5*A***). TbAAT10-1 and TbAAT2-4 were not able to support growth on any of the other amino acids tested (data not shown), and no substrate was identified for TbAAT10-2 using this approach (Fig. 5*A*). Transport assays using l-[^3^H]histidine revealed significant histidine uptake in *S. cerevisiae* cells expressing TbAAT2-4 (up to 6 min; Fig. 5*B*). In contrast, no significant uptake of l-[^3^H]histidine (up to 200 µM) was observed in TbAAT10-1-expressing cells, indicating that although TbAAT10-1 is able to transport histidine, as revealed by the growth assay (Fig. 5*A*), this amino acid is unlikely to be its preferred substrate and may be recognized only with low affinity. Analysis of histidine transport kinetics showed that TbAAT2-4 is a high-affinity histidine transporter with an apparent affinity of 20.5 ± 8.6 µM (Fig. 5*C*). Competition studies showed that TbAAT2-4-mediated histidine uptake was inhibited by several amino acids, including arginine and lysine (Fig. 5*D*). Notably, the most potent inhibitor of histidine uptake was ornithine.

**Figure 6.**
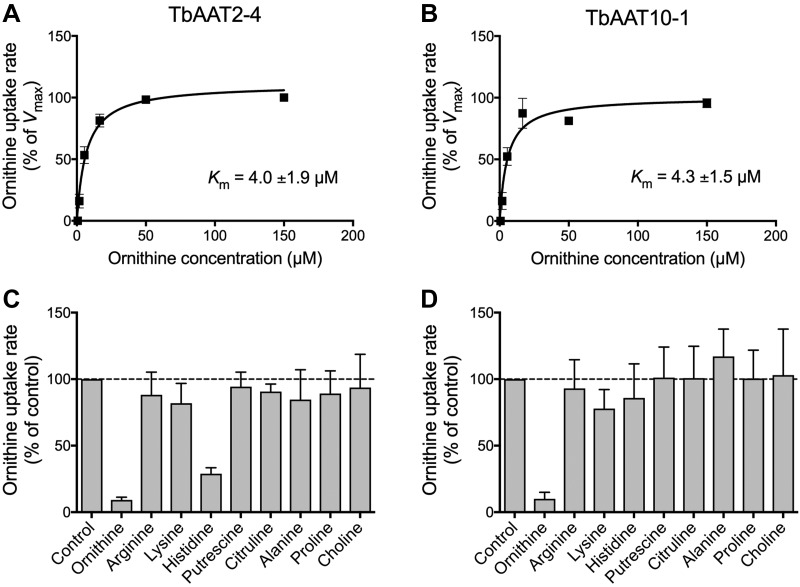
TbAAT2-4 and TbAAT10-1 are high-affinity ornithine transporters. *A*, *B*) Ornithine transport kinetics of TbAAT2-4 (*A*) and TbAAT10-1 (*B*) in *S. cerevisiae* strain 22∆8AA. Uptake rates were normalized to corresponding maximum transport rates (*V*_max_; *i.e.*, from 5.5 to 16.5 and 0.9–2.6 pmol/min/10^6^ cells for TbAAT2-4 and TbAAT10-1, respectively). Data points and apparent affinities (*K*_m_) are means ± sd from 4 independent experiments. *C*, *D*) Transport rates of *S. cerevisiae* expressing TbAAT2-4 (*C*) or TbAAT10-1 (*D*) in the presence of 50 µM ornithine and 500 µM of different compounds. Ornithine uptake rates were determined at 5 time points: 30 s and 1, 2, 3, and 5 min. The data set was normalized to control uptake rate and are means ± sd from 4 independent experiments.

In contrast to the *S. cerevisiae* complementation studies, transport assays allowed the analysis of the ornithine uptake kinetics of TbAAT2-4 and TbAAT10-1. *S. cerevisiae* expressing either TbAAT2-4 or TbAAT10-1 mediated ornithine uptake with apparent affinities of 4.0 ± 1.9 µM and 4.3 ± 1.5 µM, respectively (**Fig. 6*A, B***); no ornithine uptake was detected for TbAAT10-2 (data not shown). Competition assays demonstrated that ornithine uptake by TbAAT2-4-expressing *S. cerevisiae* was inhibited in the presence of a 10-fold excess of histidine, consistent with its high affinity for this amino acid, whereas no significant inhibition was seen after the addition of putrescine, arginine, lysine, or other amino acids (Fig. 6*C*). In contrast, ornithine transport by TbAAT10-1 was not reduced in the presence of histidine, putrescine, or any other compound tested (Fig. 6*D*). Together, these results show that TbAAT10-1 is a selective, high-affinity ornithine transporter, whereas TbAAT2-4 transports both ornithine and histidine with high affinity. The ornithine transport activity of TbAAT10-1 explained the identification of this transporter after suramin selection of the BSF *T. brucei* RNAi library. Although TbAAT2-4 was not identified by suramin selection of the BSF *T. brucei* RNAi library, our results indicate that it may also play a role in ornithine uptake by these parasites.

### TbAAT10-1 and TbAAT2-4 affect ornithine and polyamine levels in trypanosomes

**Figure 7.**
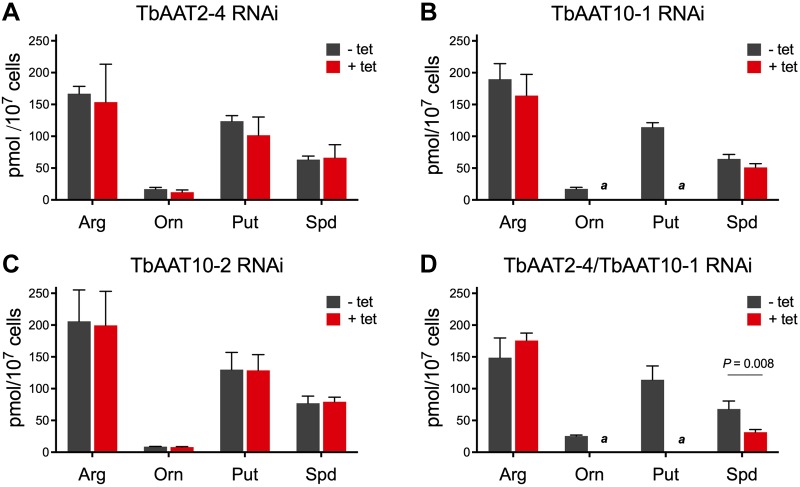
Down-regulation of TbAAT2-4 and TbAAT10-1 affects ornithine, putrescine, and spermidine levels. Amino acid and polyamine levels were determined after down-regulation of TbAAT2-4 (*A*), TbAAT10-1 (*B*), TbAAT10-2 (*C*), or TbAAT2-4/TbAAT10-1 (*D*) in BSF RNAi lines. RNAi was induced in 1 µg/ml tet for 24 h. Means ± sd from 3 technical replicates are shown. *P* values obtained by unpaired Student’s *t* test. *^a^*The analysis returned marginal or unquantifiable outputs for ornithine and putrescine after TbAAT10-1 or TbAAT2-4/TbAAT10-1 down-regulation.

The comparable affinity for ornithine of the 2 transporters when expressed in *S. cerevisiae* implies a level of redundancy for ornithine uptake in *T. brucei*, consistent with the importance of polyamine biosynthesis to the parasite. RNAi depletion of TbAAT2-4 or TbAAT10-2 had no effect on intracellular amino acid and polyamine levels (Fig. 7*A*, *C*), whereas specific down-regulation of TbAAT10-1 led to a significant reduction in intracellular ornithine and putrescine levels (Fig. 7*B*). Simultaneous depletion of TbAAT10-1 and TbAAT2-4 led to a similar reduction in intracellular ornithine and putrescine, but also resulted in a significant reduction in intracellular spermidine (Fig. 7*D*). No differences were found in the concentrations of the other amino acids tested (Supplemental Fig. 3). Together with the data on growth rates, these data support the view that TbAAT10-1 and TbAAT2-4 are the main ornithine transporters in *T. brucei*, and further highlight the reliance of *T. brucei* on exogenous ornithine for polyamine biosynthesis.

### Exogenous histidine influences TbAAT2-4-mediated ornithine uptake in BSF *T. brucei*

**Figure 8.**
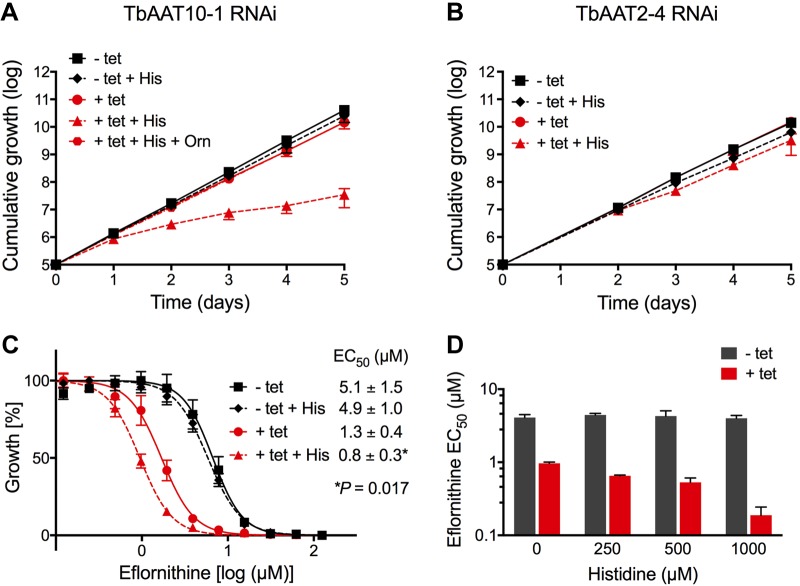
Histidine inhibits ornithine uptake by TbAAT2-4, but not by TbAAT10-1 in BSF *T. brucei*. *A*, *B*) Cumulative growth of BSF *T. brucei* after depletion of TbAAT10-1 (*A*) or TbAAT2-4 (*B*) in the absence or presence of the indicated amino acids (His, 5 mM histidine; Orn, 1 mM ornithine) in HMI-11; RNAi induced in 1 µg/ml tet. Data points are means ± sd from 2 independent clones. *C*) Representative quadruplicate EC_50_ assay showing the effect of histidine supplementation on eflornithine EC_50_ after TbAAT10-1 down-regulation in CMM (36). RNAi induced in 1 µg/ml tet 24 h before addition of eflornithine and throughout the experiment. Error bars, sem. Insets: EC_50_ values ± sd from 4 independent experiments. *P* value (+tet *vs.* +tet+his) derived from paired Student’s *t* test. *D*) Pooled EC_50_ data from 2 independent clones showing the impact of histidine supplementation on eflornithine efficacy after TbAAT10 RNAi. Error bars denote sd.

The data above indicate that TbAAT10-1 and TbAAT2-4 play redundant roles in maintaining growth (compare the growth defects in Figs. 2*A* and 4*A* and Supplemental Fig. 2). Given the affinity of TbAAT2-4 for histidine, we speculated that increasing the histidine concentration in the growth medium would reduce ornithine transport by TbAAT2-4, rendering the cells more sensitive to TbAAT10-1 knockdown. We tested this hypothesis by down-regulating TbAAT10-1 in the presence of 5 mM histidine. As predicted, growth was substantially impaired after specific TbAAT10-1 knockdown in the presence of excess histidine (Fig. 8*A*). In contrast, excess histidine had no effect on parasite growth after depletion of TbAAT2-4 (Fig. 8*B*).

The relationship of TbAAT10-1, TbAAT2-4, and histidine was further investigated in light of parasite sensitivity to eflornithine. We reasoned that eflornithine hypersensitivity observed after TbAAT10-1 depletion in BSF *T. brucei* (Fig. 4*C*) should be further enhanced by the addition of histidine, which would inhibit ornithine uptake by TbAAT2-4. Because CMM contains histidine at a concentration below that typically found in blood and in HMI-11 [∼50 µM compared with 80–130 and ∼240 µM, respectively (36, 54)], we used this medium to test our hypothesis. TbAAT10-1 down-regulation in CMM led to a significant reduction in eflornithine EC_50_ (Fig. 8*C*) that was potentiated by addition of histidine, in a dose-dependent manner (Fig. 8*D*). These data demonstrate that ornithine uptake by TbAAT2-4 is dependent on the histidine concentration in the extracellular medium and that ornithine uptake by both TbAAT10-1 and TbAAT2-4 reduce the potency of eflornithine.

## DISCUSSION

*T. brucei* is highly sensitive to perturbations in its spermidine biosynthetic pathway and to subsequent changes in polyamine and trypanothione levels. In parasitic trypanosomatids, different approaches have evolved to address their polyamine needs. These strategies reflect the levels of available metabolites in their environments. In the intracellular milieu which *Leishmania* and *T. cruzi* encounter in the mammalian host, polyamines are abundant, although most are bound to nucleic acids and proteins (16, 55). In contrast, extracellular *T. brucei* is in contact with marginal concentrations of polyamines [0.3 µM (54, 56)] and low levels of ornithine [50–100 and 4–6 µM in human plasma and cerebrospinal fluid, respectively (54)]. Unlike *Leishmania* and *T. cruzi*, *T. brucei* is not capable of high-affinity transport of polyamines, although an uncharacterized low-affinity putrescine-uptake system is present in *T. brucei*, as shown by our complementation experiments (Fig. 3) and the dispensability of ODC in the presence of exogenous putrescine (29). The absence of a high-affinity polyamine uptake system but high intracellular levels of polyamines in *T. brucei* [∼1.1 mM putrescine and ∼3.5 mM spermidine (37)], necessitates a highly active biosynthetic pathway coupled with the efficient uptake of polyamine precursors. Arginine is the precursor of polyamines in many prokaryotes and eukaryotes, but arginase activity has not been detected in *T. brucei* by conventional methods, instead metabolomic analyses provided evidence that labeled arginine is metabolized into ornithine *via* an unknown pathway (33). A growth defect observed after down-regulation of TbAAT10-1 in the presence of exogenous histidine (Fig. 8*A*), as well as a growth defect after simultaneous RNAi depletion of TbAAT10-1 and TbAAT2-4 (Fig. 2*A* and Supplemental Fig. 2), demonstrate that neither direct uptake of polyamines nor noncanonical arginase activities are sufficient to sustain growth of ornithine-depleted *T. brucei*. Thus, high-affinity ornithine uptake is crucial for this parasite.

In contrast to *T. brucei*, human cells are able to synthesize ornithine from arginine. Arginine is also a substrate for nitric oxide synthase-2, an enzyme responsible for NO production during microbial infections. In fact, pathogenic trypanosomatids are known for their ability to induce host arginase activity to evade the toxic effects of NO (57–60). In the case of *T. brucei*, evading NO does not seem the main benefit, instead, a detailed investigation of this mechanism suggested that the parasites induce arginase activity in host myeloid cells to increase ornithine availability and promote proliferation *in vivo* (61). Thus, ornithine uptake and metabolism may play a crucial role in *T. brucei* infections.

**Figure 9.**
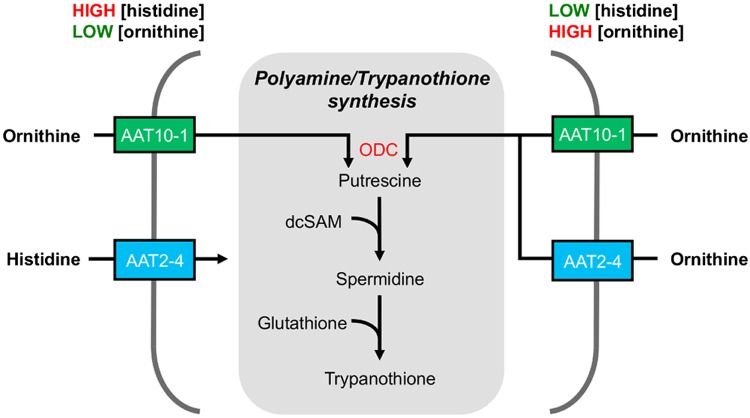
A model for ornithine uptake in *T. brucei*. Ornithine enters BSF *T. brucei* *via* 2 high-affinity transporters, TbAAT10-1 and TbAAT2-4. The contribution of TbAAT2-4 to ornithine uptake by *T. brucei* is dependent on environmental histidine concentrations.

The high-affinity ornithine transporters described in this study show how *T. brucei* is able to fulfill its high demand for ornithine, even in environments where this amino acid is scarce. To our knowledge, TbAAT10-1 and TbAAT2-4 are the first high-affinity ornithine transporters to be described in a parasite. Extracellular histidine levels influence ornithine transport by TbAAT2-4 (but not TbAAT10-1). At a high ratio of ornithine:histidine, ornithine enters the cell through both TbAAT10-1 and TbAAT2-4, whereas ornithine import *via* TbAAT2-4 is reduced at elevated histidine concentrations (**Fig. 9**). Whether this reflects a *bona fide* regulatory mechanism is unclear. Concentrations of ornithine and histidine in the blood are comparable (50–100 and 80–130 µM, respectively (54)), whereas, in cerebrospinal fluid, the ornithine concentration is lower than that of histidine [∼5 and 20 µM, respectively (54)]. In both environments, TbAAT2-4 is expected to contribute to ornithine uptake, although the negative impact of histidine may be slightly more pronounced in cerebrospinal fluid.

Studies on ornithine uptake in BSF *T. brucei* have suggested an apparent affinity for ornithine of 310 µM (33), different from the high-affinity transport mediated by TbAAT10-1 and TbAAT2-4 when expressed in *S. cerevisiae*. Although we cannot exclude an influence of the heterologous expression system, this discrepancy may also be explained by the presence of both high- and low-affinity ornithine transporters. Depending on the range of ornithine concentrations used for kinetic studies in *T. brucei*, the low-affinity transport system(s) may mask detection of high-affinity uptake systems. Our data on growth rates and intracellular concentrations of ornithine and polyamines suggest that the TbAAT10-1 and TbAAT2-4 high-affinity systems are the main ornithine transporters in *T. brucei*. Only high (nonphysiologic) concentrations of ornithine are capable of restoring parasite growth when both transporters are down-regulated, supporting the presence of transport system(s) that mediate low-affinity ornithine uptake. In *T. cruzi*, an arginine transporter (*K*_m_ for arginine of 85 µM) was shown to transport ornithine with low-affinity (*K*_m_ of 1.7 mM) (62). A contribution of such a low-affinity ornithine uptake activity to parasite growth under physiologic conditions is, however, very unlikely.

Our results demonstrate the importance of AAT10-1 and AAT2-4 to *T. brucei* polyamine homeostasis. Indeed, the loss of these transporters and the resultant impaired ornithine uptake resulted in reduced intracellular ornithine, putrescine, and spermidine levels and rendered the parasite resistant to suramin and hypersensitive to eflornithine. This result is consistent with roles for polyamine biosynthesis in suramin efficacy (19) and for ornithine supply in countering ODC inhibition and underscores the complex interplay among transport, metabolism, and drug action. Our findings not only increase knowledge on parasite physiology, but also raise the possibility that targeting ornithine uptake in *T. brucei* is a means of potentiating the therapeutic efficacy of eflornithine.

## Supplementary Material

Supplemental Data

## Abbreviations

AAT: amino acid transporter
BSF: bloodstream form
CMM: Creek’s minimal medium
FBS: fetal bovine serum
HAT: human African trypanosomiasis
NY-SM: New York single-marker
ODC: ornithine decarboxylase
qPCR: quantitative PCR
RNAi: RNA interference
SAM: *S*-adenosylmethionine
tet: tetracycline
