# Supplementary material for: Ornithine uptake and the modulation of drug sensitivity in Trypanosoma brucei
Source: FASEB J. 2017 Jul 5;31(10):4649–60. doi: 10.1096/fj.201700311R (PMC5602898; doi:10.1096/fj.201700311R)
Supplement: Supplemental Data [file supp_31_10_4649__index.html]

Ornithine uptake and the modulation of drug sensitivity in Trypanosoma brucei — Ornithine uptake and the modulation of drug sensitivity in Trypanosoma brucei — Supplemental Data 

# Ornithine uptake and the modulation of drug sensitivity in *Trypanosoma brucei*

## Supplemental Data

- Supplemental Data
- Supplemental Data
- Supplemental Data
- Supplemental Data
