# Supplementary material for: Ornithine uptake and the modulation of drug sensitivity in Trypanosoma brucei
Source: FASEB J. 2017 Jul 5;31(10):4649–60. doi: 10.1096/fj.201700311R (PMC5602898; doi:10.1096/fj.201700311R)
Supplement: Supplemental Data [file supp_fj.201700311R_Supplemental_Figure1.pdf]

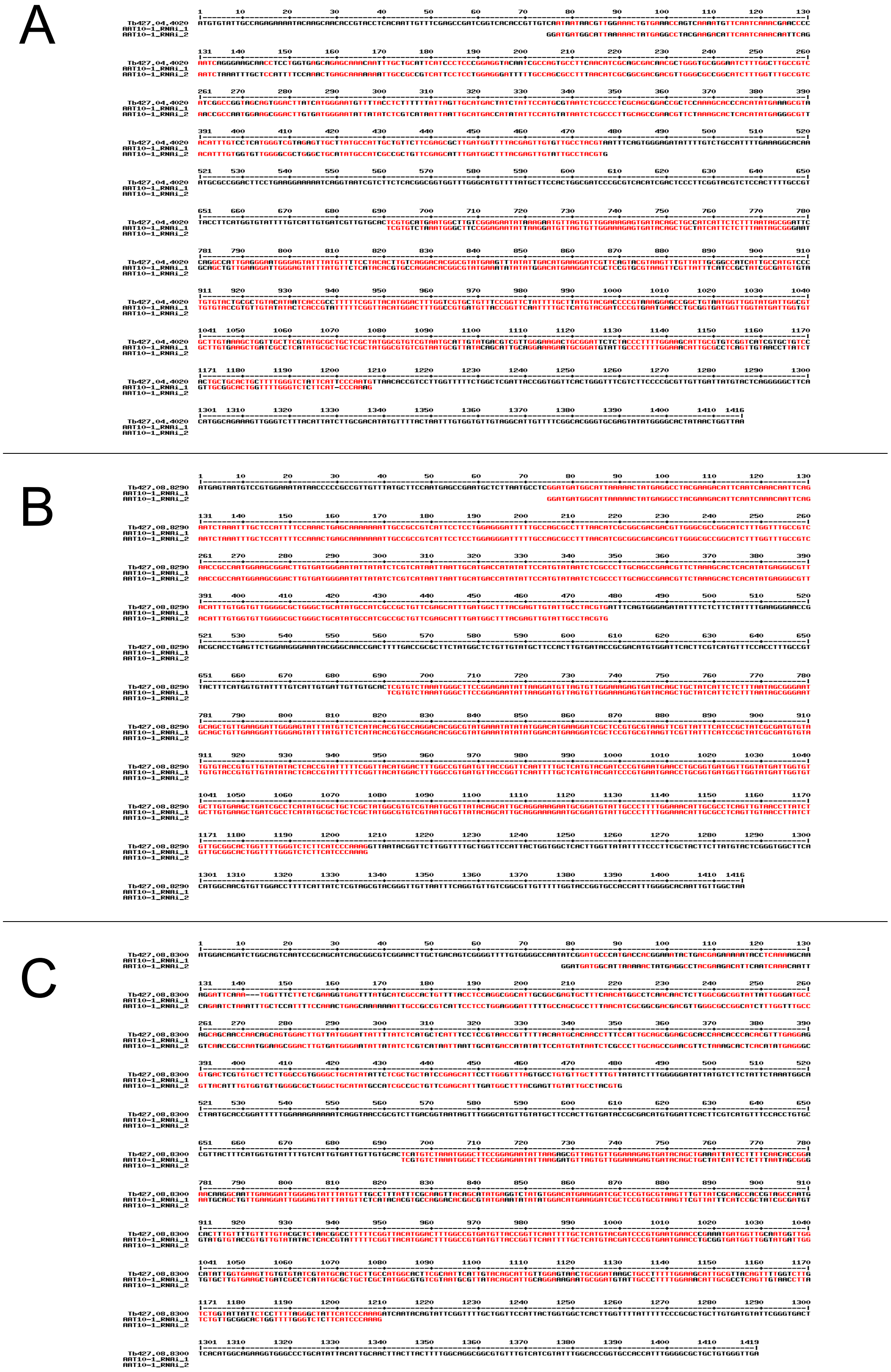

Supplementary Figure 1. Comparison of sequences of RNAi fragments with (A) TbAAT2-4 (Tb427.04.4020), (B) TbAAT10-1 (Tb427.08.8290) and (C) TbAAT10-2 (Tb427.08.8300). TbAAT10-1_RNAi_1, RNAi fragment showing both down-regulation of TbAAT10-1 and partial down-regulation of TbAAT2-4; AAT10-1_RNAi_2, RNAi fragment resulting in selective down-regulation of TbAAT10-1. The latter has a lower overall identity to TbAAT2-4 (78% instead of 82%) and shorter conserved regions.
