# Supplementary material for: Ornithine uptake and the modulation of drug sensitivity in Trypanosoma brucei
Source: FASEB J. 2017 Jul 5;31(10):4649–60. doi: 10.1096/fj.201700311R (PMC5602898; doi:10.1096/fj.201700311R)
Supplement: Supplemental Data [file supp_fj.201700311R_Supplemental_Figure2.pdf]

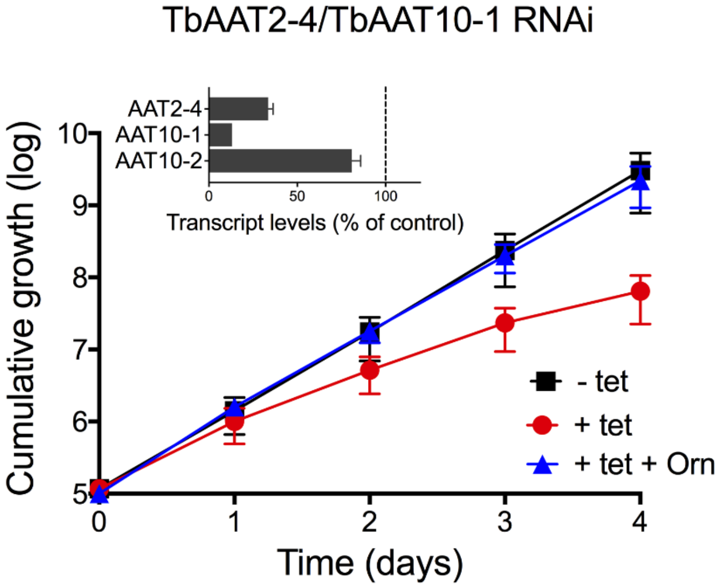

Supplementary Figure 2. Simultaneous down-regulation of TbAAT2-4 and TbAAT10-1 leads to a growth arrest of BSF *T. brucei* that can be rescued by ornithine. Cumulative growth of BSF *T. brucei* following down-regulation of TbAAT2-4 and TbAAT10-1 (double RNAi) in the presence or absence of 1 mM ornithine (Orn); RNAi induced in 1 µg ml^{-1} tetracycline. Data points are mean values ± SD from two independent clones. Inset, transcript levels determined by qRT-PCR, shown as percentage of uninduced cells and normalized to telomerase reverse transcriptase expression. Mean values ± SEM from two independent clones are shown.
