# Supplementary material for: Ornithine uptake and the modulation of drug sensitivity in Trypanosoma brucei
Source: FASEB J. 2017 Jul 5;31(10):4649–60. doi: 10.1096/fj.201700311R (PMC5602898; doi:10.1096/fj.201700311R)
Supplement: Supplemental Data [file supp_fj.201700311R_Supplemental_Figure3.pdf]

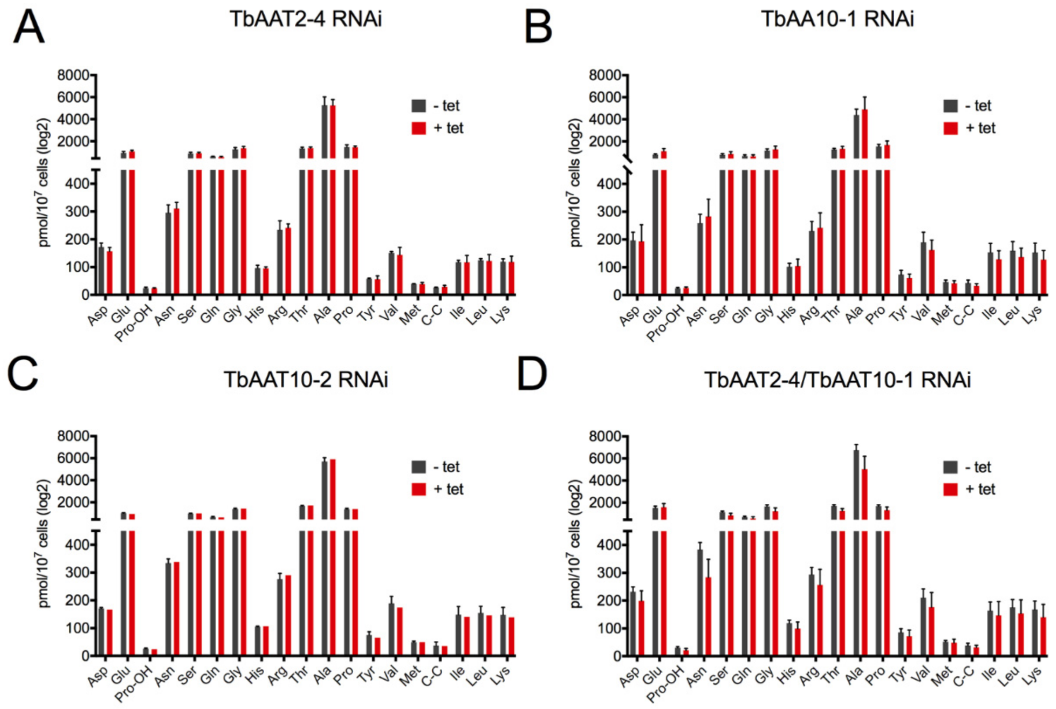

Supplementary Figure 3. Amino acid levels are comparable in induced and non-induced TbAAT2-4, TbAAT10-1, TbAAT10-2 and TbAAT2-4/TbAAT10-1 *T. brucei* BSF RNAi lines. Amino acid levels were determined one day following down-regulation of TbAAT2-4 (*A*), TbAAT10-1 (*B*), TbAAT10-2 (*C*), or TbAAT2-4/TbAAT10-1 (*D*) in BSF RNAi lines (1 µg ml^{-1} tetracycline). Mean values ± SD from three technical replicates are shown, except for TbAAT10-2 (one replicate shown). Comparable results were found when samples were analysed using the polyamine quantification method and a representative amino acid (arginine) is shown in Fig. 7. Pro-OH, hydroxyproline; C-C, cystine.
