# Supplementary material for: Ornithine uptake and the modulation of drug sensitivity in Trypanosoma brucei
Source: FASEB J. 2017 Jul 5;31(10):4649–60. doi: 10.1096/fj.201700311R (PMC5602898; doi:10.1096/fj.201700311R)
Supplement: Supplemental Data [file supp_fj.201700311R_Supplemental_Tables.pdf]

Supplementary Table S1. Closest homologs of TbAAT10-1. BLAST analysis of TbAAT10-1 (Tb927.8.8290) amino acid sequence revealed higher identity to a member of the TbAAT2 subgroup, Tb427.04.4020 (TbAAT2-4).

**Gene ID**	**AAT nomenclature [52]**	**Score**	**% Identity**	**% Similarity**	**E value**
Tb927.8.8290	AAT10	950	100	100	0
Tb927.4.4020	AAT2	705	73.461	83.65	0
Tb927.8.8300	AAT10	615	69.655	83.91	0
Tb927.8.7670	AAT7	607	63.341	78.96	0
Tb927.8.7600	AAT7	603	62.208	78.56	0
Tb927.8.7650	AAT7	603	64.502	79.87	0
Tb927.4.4010	AAT2	594	59.789	77.26	0
Tb927.4.3990	AAT2	593	59.789	77.26	0
Tb927.4.4000	AAT2	593	59.789	77.26	0
Tb927.4.4870	AAT4	586	64.444	80.89	0
Tb927.8.7630	AAT7	584	62.801	75.93	0
Tb927.8.7620	AAT7	583	62.582	75.93	0
Tb927.4.4830	AAT4	573	63.616	79.91	0
Tb927.4.4850	AAT4	573	63.616	79.91	0
Tb927.8.7610	AAT7	568	61.688	75.54	0
Tb927.8.7640	AAT7	568	60.934	74.73	0
Tb927.8.7680	AAT7	469	50.955	68.79	1.31E-162
Tb927.8.7700	AAT7	463	50.531	68.37	2.19E-160

Supplementary Table S2. Nucleotide identity of TbAAT10-1 (Tb427.08.8290) to the closest *T. brucei* homologs identified by BLAST and ClustalO analysis.

**Gene ID**	**AAT nomenclature [52]**	**% Identity to TbAAT10-1**
Tb927.8.8290	TbAAT10-1	100
Tb927.4.4020	TbAAT2-4	74.72
Tb927.8.8300	TbAAT10-2	70.68
